# Zinc phosphate-based nanoparticles as alternatives to zinc oxide in diet of weaned piglets

**DOI:** 10.1186/s40104-020-00458-x

**Published:** 2020-06-09

**Authors:** Silvia Kociova, Kristyna Dolezelikova, Pavel Horky, Sylvie Skalickova, Daria Baholet, Lucie Bozdechova, Eva Vaclavkova, Jaroslava Belkova, Pavel Nevrkla, Jiri Skladanka, Tomas Do, Ondrej Zitka, Yazan Haddad, Pavel Kopel, Ludek Zurek, Vojtech Adam, Kristyna Smerkova

**Affiliations:** 1 Department of Chemistry and Biochemistry, Mendel University in Brno, Zemedelska 1, CZ-613 00 Brno, Czech Republic; 2 Central European Institute of Technology, Brno University of Technology, Purkynova 123, CZ-612 00 Brno, Czech Republic; 3 Department of Animal Nutrition and Forage Production, Mendel University in Brno, Zemedelska 1, CZ-613 00 Brno, Czech Republic; 4 Institute of Animal Science, Pratelstvi 815, CZ-104 00 Praha Uhrineves, Czech Republic; 5 Department of Animal Breeding, Mendel University in Brno, Zemedelska 1, CZ-613 00 Brno, Czech Republic; 6 Department of Inorganic Chemistry, Faculty of Science, Palacky University, 17. listopadu 12, CZ-771 46 Olomouc, Czech Republic; 7 Central European Institute of Technology, Center for Zoonoses, University of Veterinary and Pharmaceutical Sciences, Brno, Palackeho 1946/1, CZ-612 42 Brno, Czech Republic

**Keywords:** Antioxidant status, Diet, *E. coli* STa, STb, Stx2, F4, F18, Fecal coliforms, Microbiota

## Abstract

**Background:**

The high doses of zinc oxide (ZnO) administered orally to piglets for the prevention of diarrhea and increase of growth rate can contaminate pig farms and the surrounding environment. Therefore, there is a need to find a replacement of high doses of dietary ZnO with an equally effective alternative. In the present study, the effect of two formulations of zinc phosphate-based nanoparticles (ZnA and ZnC NPs) on growth performance, intestinal microbiota, antioxidant status, and intestinal and liver morphology was evaluated. A total of 100 weaned piglets were randomly divided into 10 equal groups with the base diet (control) or the base diet supplemented with ZnA, ZnC, or ZnO at concentrations 500, 1000, and 2000 mg Zn per kilogram of diet. Supplements were given to animals for 10 days. Fecal samples were collected on day 0, 5, 10 and 20. At the end of the treatment (day 10), three piglets from each group were sacrificed and analyzed.

**Results:**

Comparing to that of control, the significantly higher piglet weight gain was observed in all piglet groups fed with ZnA (*P* < 0.05). Differences in the total aerobic bacteria and coliform counts in piglet feces after NPs supplementation compared to that of control and ZnO groups were also found (*P* < 0.05). The majority of aerobic culturable bacteria from the feces represented *Escherichia* (28.57–47.62%), *Enterococcus* (3.85–35.71%), and *Streptococcus* (3.70–42.31%) spp. A total of 542 *Escherichia coli* isolates were screened for the virulence genes *STa*, *STb*, *Stx2*, *F4*, and *F18*. The substantial occurrence of *E. coli* virulence factors was found on day 5, mainly in fimbrillary antigen and thermostable toxins, except for piglets fed by ZnC. Zn treatment decreased Zn blood levels in piglets fed with ZnO and ZnA (500 mg/kg) and increased in ZnC (2000 mg/kg) compared to that of control (*P* < 0.05). The antioxidant status of piglets was affected only by ZnA. While some changes in the liver and the intestinal morphology of piglets with NPs were observed, none were serious as reflected by the normal health status and increased weigh gain performance.

**Conclusions:**

Our results indicate that ZnA NPs have a positive effect on the piglet growth performance even at the lowest concentration. The prevalence of *E. coli* virulence factors was lowest in pigs supplemented with ZnC. Zinc phosphate-based nanoparticles may be an effective alternative to ZnO.

## Background

Zinc is an essential trace element for animals as it plays an important role in nutrition, growth, and immunity. Due to its efficiency and a reasonable price, zinc in the form of zinc oxide (ZnO) has been commonly used in high doses (2000 to 3000 mg/kg diet) for weaned piglets as an alternative to antibiotics to prevent intestinal inflammation and increase weight gain [1, 2]. However, starting in 2022 in the European Union, zinc use in such high concentrations will be banned [3, 4]. This is because zinc requirements for piglets are only 80 to 100 mg/kg [5] and therefore high medication doses of zinc are not utilized by the animals and can result in environmental contamination [6, 7]. In fact, it has been shown that pig farm sites have high concentrations of zinc in the soil [8]. Moreover, the high zinc doses (2500 mg/kg diet) affect the intestinal microbiota, and there is also evidence for co-selection of the antibiotic resistance traits [9, 10].

Zinc nanoparticles (ZnNPs) can be an excellent alternative to high doses of zinc, in terms of higher pharmacokinetic efficiency, especially when used against coliform bacteria in other mammals [11]. ZnNPs (450 mg/kg diet) significantly reduced the *Escherichia coli* population in the small and large intestine in weaned piglets. Furthermore, it lead to much lower excretion of zinc in the feces in comparison to that of the same dose of ZnO [12]. It has been demonstrated that ZnNPs in pig nutrition can reduce the zinc use up to 60% while maintaining the same positive effect on the intestinal microbiome [13]. Nevertheless, there are many questions remaining about the safety of nanoparticles and their metabolites for animals and their fate and the effect on the environment. For example, it has been shown that some nanoparticles may impair the growth of aquatic plants [14]. Zinc is also a strong antioxidant related to the protection of porcine dermis and its derivatives [15, 16].

Based on our previous work, where four types of ZnNPs were characterized and tested on rats [11], the two types of antibacterial zinc phosphate-based NPs were studied as alternatives to ZnO in the diet of weaned piglets. ZnNPs positively influenced the growth performance of piglets compared to that of control. Moreover, the effect of ZnNPs on the intestinal microbiome, antioxidant markers, intestinal and liver morphology, and the diarrhea occurance of growing pigs was evaluated.

## Materials and methods

### Chemicals

All chemicals were purchased from Sigma Aldrich (St. Louis, MO, USA) and Penta (Prague, Czech Republic) of p.a. purity, unless noted otherwise. The pH was measured using inoLab Level 3 (Wissenschaftlich-Technische Werkstatten GmbH; Weilheim, Germany). Deionised water underwent demineralisation by reverse osmosis using the instruments Aqua Osmotic 02 (Aqua Osmotic, Tisnov, Czech Republic) and then it was subsequently purified using Millipore RG (Millipore Corp., Waltham, MA, USA) – 18 MΏ MilliQ water.

### ZnNPs synthesis

The phosphate-based ZnNPs were synthesized according to the procedure published in our previous study [11]. Briefly, ZnA NPs were prepared by dissolving of Zn(NO_3_)_2_·6H_2_O (44.6 g) in 500 mL of milliQ water and heating to 60 °C. Then, 13.2 g of (NH_4_)_2_HPO_4_ dissolved in 200 mL of milliQ water was added. After 2 h of stirring, the mixture was made up to 1000 mL with milliQ water. The ZnC NPs were prepared by dissolving of Zn(NO_3_)_2_·6H_2_O (30 g) in 500 mL of milliQ water and heating to 60 °C. Then, 13.3 g Na_4_P_2_O_7_ dissolved in 200 mL of milliQ water was added. After 2 h of stirring, the mixture was made up to 1000 mL with milliQ water.

### Animal experiment

The experiment was performed with the approval of the Ethics Committe at the Faculty of AgriSciences, Mendel University in Brno, Czech Republic in accordance with the Act No. 246/1992 Coll. on the protection of animals against cruelty.

Proposed experiment was conducted on an accredited experimental farm of the Research Institute of Animal Production in Prague (Czech Republic). The experiment was carried out on weaned piglets divided into 10 groups with 10 animals in each group. The sex ratio in the group was 50:50 (castrates:females). The first group served as control, where zinc intake was not manipulated in the diet. The second, third, and fourth group were supplemented with zinc in the form of ZnO at a dose of 500, 1000, and 2000 mg of Zn equivalent per kilogram of diet, respectively. The fifth, sixth and seventh groups of piglets were supplemented with ZnA nanoparticles at a dose of 500, 1000 and 2000 mg of Zn equivalent per kilogram of diet, respectively. The last tested supplementation was ZnC nanoparticles, which were added to the eighth, ninth, and tenth group of piglets at doses of 500, 1000 and 2000 mg of Zn equivalent per kilogram of diet, respectively.

The experiment started on the weaning day of the piglets (day 28 of the animal age), and the Zn feeding lasted for 10 days. Different forms of zinc were mixed into the piglet mixture (Table 1 and Table S1). The total content of phosphorus in the diet was 6.49 g/kg —calculated by the feed program Agrokonzulta (Czech Republic). The piglets had a feed and water available *ad libitum*. The animal husbandry complied with Decree No. 208/2004 Coll. (Decree on minimum standards for the protection of farm animals).

**Table 1:** Composition of the diet

Component	Quantity, %
Barley seed	41.2
Grain selvico test	25.0
Wheat seed	17.4
Dried poultry blood	4.5
Trace mineral-vitamin premix^a^	3.8
Wheat bran	3.0
Fish meal 70%	2.6
Molcolac	1.0
Formic acid	1.0
Fish fat	0.53

^a^Supplied per kilogram diet as feed basis: vitamin A, 5000 IU; vitamin D_3_, 800 IU; vitamin E, 30 IU; vitamin K_3_, 1.0 mg; biotin, 0.05 mg; folic acid, 0.3 mg; niacin, 10 mg; *D*-pantothenic acid, 10 mg; riboflavin, 3.6 mg; thiamine, 1.0 mg; pyridoxin, 1.5 mg; choline, 800 mg; Zn (ZnSO_4_), 120 mg; Fe (FeSO_4_), 125 mg; Cu (CuSO_4_·5H_2_O), 15 mg/kg; Mn (MnSO_4_·H_2_O), 10 mg/kg; I (KI), 0.15 mg; Se (Na_2_SeO_3_), 0.2 mg; enramycin, 20 mg; chlortetracycline, 50 mg

Before starting the experiment, piglets were weighed, and 10 mL of blood from *vena jugularis externa* was collected and aliquotted in two tubes by a veterinarian. At the end of the feeding stage of the experiment (10 days), body weight and blood collection were conducted as described above. Three piglets (females) were sacrificed from each group, and the liver and the small intestine (1/3 of the duodenum) were collected for histological analysis. Generally, females have more sensitive antioxidant system than males. Moreover, females are more susceptible to acute liver injury from xenobiotics than males [17–19]. The samples were fixed in a 10% formalin solution. The sacrificing of piglets was performed with the Exagon® anaesthetic (400 mg/mL). Piglets were given general anesthesia with a combination of Xylazine® and Ketamine®. Remaining piglets were weighed again at the end of the experiment on day 20.

### Quantitative and qualitative analysis of the microbiome in piglet feces

Fresh fecal samples were collected from individual piglets immediately before the start of the zinc treatment (day 0) and then on day 5, 10 and 20. The fresh feces were sampled into sterile collection tubes, kept on wet ice and processed within 4 h. Determination of the total aerobic microorganisms and the total coliforms count was performed. In addition, qualitative identification of the major bacterial groups was conducted. Both analyses were carried out separately for each sample. The fecal samples were homogenized in sterile 0.85% saline (1:9 *w/v*), and the homogenate was then serially diluted. Subsequently, 1.0 mL of diluted suspension was pour plated on the Plate Count agar (PCA) and MacConkey agar (Sigma-Aldrich) in duplicates. All colonies from PCA and from MacConkey agar were counted after 24 h at 37 °C. The results are expressed as log(CFU/g) of feces. For identification of the major groups of bacteria, the fecal samples were applied on selective agars designed for growth of *E. coli* and coliforms (HiCrome Chromogenic Coliform HiCynth agar, Himedia, India), streptococci, and enterococci (Columbia blood agar with Streptococcus selective supplement, Oxoid, UK; Mitis Salivarius agar, Sigma-Aldrich). Representative individual colonies were picked from each agar plate and streaked on 5% blood sheep agar and incubated at 37 °C for 24 h. Individual isolates were identified by the matrix-assisted laser desorption/ionization time-of-flight mass spectrometer (MALDI-TOF MS) Bruker ultrafleXtreme (Bruker Daltonik GmbH, Bremen, Germany) using MALDI BioTyper™ Compass Explorer 4.1.90 analysis software equipped with MBT 8468 MPS library. Bacterial isolates were then stored at − 80 °C for further analysis.

### *E. coli* virulence factors

Confirmed *E. coli* isolates were screened for five virulence factors (*F18*, *F4*, *STa*, *STb*, *Stx2*) and one house-keeping gene (*uidA*) by multiplex PCR. Briefly, a loopfull of each isolate was transferred to 200 μL of sterile distilled water in a 1.5 mL sterile microcentrifuge tube. Cells were lysed by boiling for 10 min, incubated on ice for 5 min, and centrifuged for 2 min at 13500×*g*. Three microliters of the supernatant were used per PCR reaction. The following *E. coli* strains were used as positive controls: *E. coli* 13502 (for *Stx2* and *F18*), *E. coli* 10423 (for *STa*, *STb,* and *F18*) and *E. coli* 11803 (for *STb* and *F4*). *E. coli* 7929 (from the Czech Collection of Microorganisms, Brno) was used as a non-pathogenic (negative) control. The mixture for PCR reaction (25 μL) was composed of 12.5 μL Q5 Hot Start High Fidelity 2X Master Mix (NEB, USA), 0.3 μL of primers for *Stx2* (*VT2,* 10 μmol/L) and *F18* (10 μmol/L), 0.46 μL of *STb* primers (10 μmol/L) and 0.63 μL of *STa* primers (10 μmol/L), 6.14 μL of deionized water and 3 μL of lysate. The second reaction was performed for *F4* and *uidA*. The mixture for PCR reaction (25 μL) was composed of 12.5 μL Q5 Hot Start High Fidelity 2X Master Mix (NEB, USA), 0.625 μL of *F4* primers (10 μmol/L), 1 μL of *uidA* primers (10 μmol/L), 6.25 μL of deionized water and 3 μL of lysate. Primers used in this study are described in Table 2. Amplification was done on a Mastercycler nexus (Eppendorf, Germany). Cycling conditions were as follows: 90 °C for 5 min, 35 cycles of 90 °C for 1 min, 61 °C for 1 min, 72 °C for 1 min, followed by 72 °C extension for 10 min and 4 °C hold. The PCR products were visualized by gel electrophoresis using 1.5% agarose (Sigma Aldrich, USA).

**Table 2:** Primers used virulence factor detection

Virulence factor	Primer sequence (5′→3′)	PCR product size, bp	Reference
F18	TGGTAACGTATCAGCAACTA	313	[20]
	ACTTACAGTGCTATTCGACG		
F4	GCCTGGATGACTGGTGATTT	709	[21]
	TCTGACCGTTTGCAATACCC		
STa	CAACTGAATCACTTGACTCTT	158	[20]
	TTAATAACATCCAGCACAGG		
STb	TGCCTATGCATCTACACAAT	113	[20]
	CTCCAGCAGTACCATCTCTA		
Stx2 (VT2)	GTTTTTCTTCGGTATCCTATTCC	484	[22]
	GATGCATCTCTGGTCATTGTATTAC		
uidA	TGTTACGTCCTGTAGAAAGCCC	153	[23]
	AAAACTGCCTGGCACAGCAATT		

### Glutathione peroxidase analysis

The activity of glutathione peroxidase (GPx) was carried out according to the protocol of RANSEL (Randox, United Kingdom). Briefly, GPx catalyzed the glutathione (GSH) oxidation by peroxide to its oxidized form (GSSG). In the presence of glutathione reductase and NADPH, GSSG is converted to GSH and simultaneously the NADPH is oxidized to NADP+ which is accompanied by a change in absorbance maximum. The analysis was carried out on the spectrophotometer Secomam S.500P (Secomam, USA) with absorbance wavelength set to 340 nm.

### Malondialdehyde analysis

Determination of malondialdehyde (MDA) was carried out using the spectrophotometer Agilent Cary 60 (Agilent, USA). MDA is one of the secondary products of lipid peroxidation. At elevated temperature, a red complex with thiobarbituric acid (TBA) forms in an acidic environment. The absorbance of the resulting color complex MDA-TBA is measured at three wavelengths of 485 nm, 532 nm, and 560 nm.

### Zinc detection

Zinc concentration was determined by the atomic absorption spectrometer with flame and electrothermal atomization Varian AAS DUO 280 Z + 240 FS – FAAS flame technology (Agilent, USA).

### Statistical analysis

The bacterial counts were analysed using IBM SPSS Statistics 21 (IBM Corporation, Armonk, New York, USA) and the differences with *P* < 0.05 were considered as significant. Zinc concentration (0, 500, 1000 and 2000 mg/kg), treatment group (ZnA, ZnC and ZnO) and time period (0, 5, 10 and 20 days) were treated as categorical data types throughout all tests. Extreme (far) outliers of the total and coliform counts were removed according to boxplots of treatment groups and Zn concentration in each time period. Extreme outliers were values outside the 3× interquartile range, which most likely resulted from errors in pipetting and dilutions. Near outliers (outside the 1.5× interquartile range) were retained.

In each Zn treatment group, analysis of variance (ANOVA) test was used to detect significant differences in microbial growth based on Zn concentrations. Pairwise comparisons based on the weighted average of “Studentized” ranges were done using the Dunnett’s C *post hoc* test for unequal variances. The paired T-test was used to study significant differences in microbial growth between time periods and day zero.

The comparison between treatment groups and ZnO group was performed using independent sample T-test and Mann Whitney test. Due to the frequent significance in Levene test of equal variances and inconsistencies in T-test results, the non-parametric Mann Whitney test results were considered more reliable in this part of the study.

To study the significance of Zn treatments and dosage as risk factors for diarrhea, adjusted odds ratios (OR) were calculated by binary logistic regression. The event of diarrhea was used as dependent variable with binary 0/1 values. Covariates were added via a×b entry method to calculate cross-adjusted OR. Confidence interval (CI) for OR were calculated at 95%.

The data for antioxidant status and Zn levels were analyzed using STATISTICA.CZ, version 12.0 (Czech Republic). The results were expressed as a mean from all samples ± standard deviation. Statistical significance was determined by examining the basic differences among groups using ANOVA and Scheffé’s method for all parameters. The differences with *P < *0.05 were considered as significant.

## Results

### Piglet growth

Piglets had a mean weight between 6.0 and 8.1 kg in different groups at the start of the experiment (Table 3). The control group had a weight gain of 2.0 kg after 20 days. At a dose of 500 mg Zn/kg diet, the highest weight increase was observed in the group of piglets with ZnA NPs (3.7 kg). Piglets fed a Zn dose of 1000 mg/kg diet had the highest weight increase in the ZnA group (3.7 kg). At the dose of Zn 2000 mg/kg diet, the highest weight increase was also observed in the ZnA group (4.4 kg). Overall, significantly higher weight gains (*P* < 0.05) were observed in ZnA piglet groups compared to that of the control group at all Zn concentrations. The increase was 2.5 kg, 2.7 kg, and 3.4 kg for Zn dose 500, 1000, and 2000 mg Zn/kg diet, respectively. Also, group ZnC (dose 2000 mg/kg) and ZnO (1000 mg/kg) showed a significant increase (*P* < 0.05) in weight compared to that of control. Significant differences in weight gain were not observed between groups supplemented with zinc nanoparticles (ZnA, ZnC) and ZnO. Body weight gain was highest in the ZnA 2000 mg/kg group (4.4 kg). The lowest body weight gain was observed in the control group (2.0 kg).

**Table 3:** Piglet weight during the experiment

Day	Control	500 mg/kg			1000 mg/kg			2000 mg/kg		
		ZnO	ZnA	ZnC	ZnO	ZnA	ZnC	ZnO	ZnA	ZnC
0	6.6 ± 1.3	7.1 ± 0.4	7.4 ± 0.9	6.0 ± 0.9	7.9 ± 1.2	7.6 ± 1.7	6.8 ± 1.8	7.0 ± 0.9	7.6 ± 1.5	8.1 ± 0.9
10	7.7 ± 1.0	8.6 ± 1.1	8.6 ± 1.1	8.0 ± 1.1	9.0 ± 1.4	7.5 ± 1.3	9.1 ± 1.7	8.6 ± 1.1	8.9 ± 1.5	10.3 ± 0.9*
20	8.6 ± 1.5	9.9 ± 1.1	11.1 ± 0.9*	9.2 ± 1.1	11.5 ± 0.9*	11.3 ± 1.0*	9.8 ± 1.0	9.9 ± 1.3	12.0 ± 1.8*	10.9 ± 1.0
WG	2.0 ± 0.7	2.8 ± 1.4	3.7 ± 0.8*	3.2 ± 0.7	3.6 ± 0.8*	3.7 ± 1.6	3.0 ± 0.8	2.9 ± 0.9	4.4 ± 1.0*	2.8 ± 0.9

*WG* weight gain (difference between average weight on day 0 and day 20)

*indicates significant differences (*P* < 0.05) between the control and the treated group

### Effect of Zn treatments on aerobic bacteria and coliforms counts

**Fig. 1:**
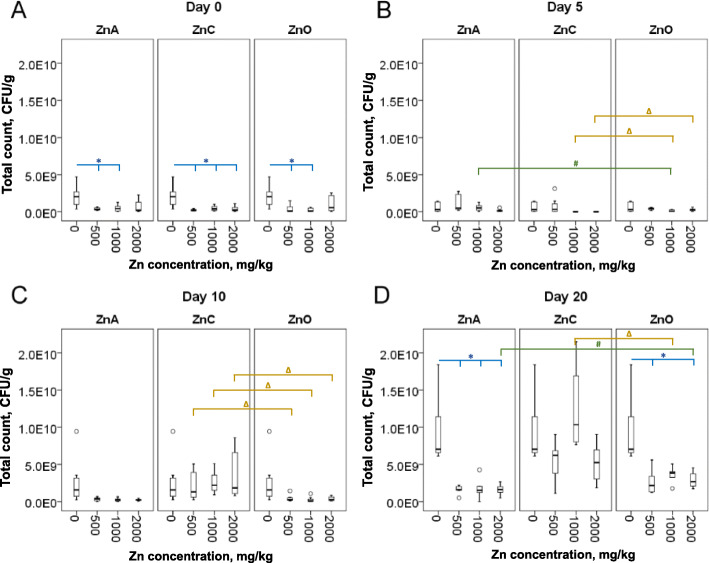
Total counts of aerobic bacteria at day 0 (**a**) and after 5 days (**b**), 10 days (**c**) and 20 days (**d**) of Zn treatment. Boxes represent Q1 and Q3 quartiles with median bar while T-whiskers represent 95% confidence intervals of 6–10 individuals. * indicates significant differences (*P* < 0.05) between group without treatment (0) and groups with Zn treatment. # indicates significant differences (*P* < 0.05) between ZnA and ZnO group (the equal Zn concentrations were compared). ∆ indicates significant differences (*P* < 0.05) between ZnC and ZnO group (the equal Zn concentrations were compared)

The counts of total aerobic bacteria determined in piglet feces over 20 days are shown in Fig. 1. The overall average counts were 7.71 × 10^8^ CFU/g feces for ZnA, 2.76 × 10^9^ CFU/g for ZnC, 1.06 × 10^9^ CFU/g for ZnO, and 4.28 × 10^9^ CFU/g for the control group. Compared with the control group, the significant difference (*P* < 0.05) was determined for ZnA (500 and 1000 mg/kg), ZnC (500, 1000, and 2000 mg/kg), and ZnO (500 and 1000 mg/kg) at day 0 (Fig. 1a). Then, the significant decrease (*P* < 0.05) was observed at day 20 (Fig. 1d) in the case of ZnA (500, 1000, and 2000 mg/kg) and ZnO (500 and 2000 mg/kg) compared with the control group. When comparing the NPs treatment with ZnO, the significant mean difference was found between ZnA and ZnO at day 5 (1000 mg/kg) and day 20 (2000 mg/kg) when ZnA counts were significantly higher (*P* = 0.021) on 5^th^ day and conversely significantly lower (*P* = 0.026) on 20^th^ day (Fig. 1b, d). Between ZnC and ZnO, the significant differences were found at day 5, 10 and 20 (Fig. 1b, c, d). Specifically, ZnC (1000 and 2000 mg/kg) showed a significant decrease (*P* < 0.001) in counts at day 5, while on the 10^th^ day of treatment, all concentrations of ZnC caused significantly increased bacterial growth (*P* < 0.01). The significantly higher (*P* = 0.01) counts for ZnC (1000 mg/kg) were also observed on day 20 compared to ZnO.

**Fig. 2:**
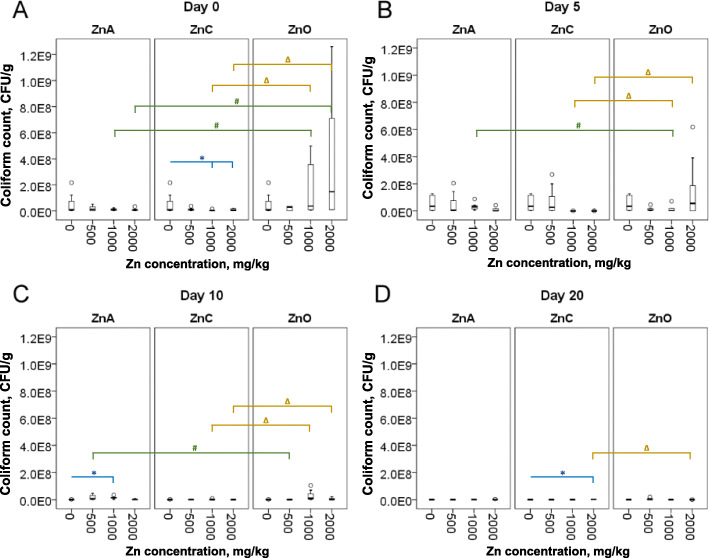
Counts of coliforms at day 0 (**a**) and after 5 days (**b**), 10 days (**c**) and 20 days (**d**) of Zn treatment. Boxes represent Q1 and Q3 quartiles with median bar while T-whiskers represent 95% confidence intervals of 6–10 individuals. * indicates significant differences (*P* < 0.05) between group without treatment (0) and groups with Zn treatment. # indicates significant differences (*P* < 0.05) between ZnA and ZnO group (the equal Zn concentrations were compared). ∆ indicates significant differences (*P* < 0.05) between ZnC and ZnO group (the equal Zn concentrations were compared)

The change of coliforms counts in piglet feces during the treatment is presented in Fig. 2. The overall mean coliforms counts were calculated as 1.36 × 10^7^ CFU/g feces for ZnA, 8.71 × 10^6^ CFU/g for ZnC, 6.42 × 10^7^ CFU/g for ZnO, and 7.76 × 10^7^ CFU/g for the control group. The coliform concentration was significantly lower (*P* < 0.05) in ZnC group (1000 and 2000 mg/kg) at day 0 compared to the control (Fig. 2a). Interestingly, the significant increase (*P* < 0.05) of coliforms was observed in ZnA (1000 mg/kg, day 10) and ZnC (2000 mg/kg, day 20) groups in comparison with the control (Fig. 2c, d). When comparing ZnO with NPs treatment, the difference in coliforms growth was significant on all collection days. The coliforms counts were significantly higher for ZnO group in comparison to that in ZnA group 1000 (*P* = 0.035) and 2000 mg/kg (*P* = 0.002); and also ZnC group 1000 (*P* = 0.001) and 2000 mg/kg (*P* = 0.003) at day 0 (Fig. 2a). In contrast, on day 5 (Fig. 2b), the coliform concentration significantly increased (*P* = 0.027) for ZnA (1000 mg/kg). ZnC treatment (1000 and 2000 mg/kg) caused a significant decrease (*P* < 0.05) of the coliforms concentration at day 5 of treatment compared to that of ZnO. At day 10 of the treatment (Fig. 2c), the similar pattern to that on day 5 was found. ZnA (500 mg/kg) exhibited significant increase (*P* = 0.017) of coliforms; however, the counts significantly decreased (*P* < 0.01) in the case of ZnC treatment (1000 and 2000 mg/kg). And vice versa, compared to ZnO the coliforms growth was significantly higher (*P* = 0.014) at day 20 (Fig. 2d) for ZnC (2000 mg/kg).

The temporal comparison showed significant changes between day 0 and the rest of the days in all treatments (Fig. S1, note: controls were removed from paired T-test). During ZnA treatment (Fig. S1A, B), paired-mean total CFUs was significantly lower (*P* = 0.038) in day 10 compared to day 0 by 2.79 × 10^8^ CFUs. Whereas paired-mean total CFUs was significantly higher (*P* < 0.001) on day 20 compared to day 0 by 1.30 × 10^9^ CFUs. Paired-mean coliform counts were significantly lower (*P* = 0.003) in day 20 compared to day 0 by 1.23 × 10^7^ CFUs. Moreover, significant positive moderate correlation (*R*^2^ = 0.449, *P* = 0.021) was observed in coliforms between day 0 and day 5. After ZnC treatment (Fig. S1C, D) paired-mean total counts were significantly higher (*P* < 0.001) on day 10 compared to day 0 by 2.25 × 10^8^ CFUs. The significantly higher (*P* < 0.001) counts were also observed in day 20 compared to day 0 by 6.72 × 10^9^ CFUs. In contrast, the coliforms counts were significantly lower (*P* < 0.001) on day 10 compared to day 0 by 7.61 × 10^6^ CFUs and significant reduction (*P* = 0.005) was found on day 20 compared to day 0 by 7.65 × 10^6^ CFUs. In ZnO treatment (Fig. S1E, F) paired-mean total counts were significantly higher (*P* < 0.001) on day 20 compared to day 0 by 2.53 × 10^9^ CFUs. Coliform counts were significantly lower (*P* = 0.03) on day 5 compared to day 0 by 1.60 × 10^8^ CFUs and significantly lower (*P* = 0.006) as well on day 10 compared to day 0 by 2.25 × 10^8^ CFUs.

### Zn treatment and dosage as risk factors for piglet diarrhea

To further investigate the role of Zn treatments and dosage as risk factors for piglet diarrhea, binary logistic regression was used for calculating cross-adjusted OR. Interestingly, the increase in log_10_CFU of coliforms (cross-adjusted with either Zn treatment or concentration) was significantly more associated with diarrhea events in most cases compared to total counts log_10_CFU (see Additional file Table S2).

Based on Zn concentrations, total counts log_10_CFU cross-adjusted with 1000 and 2000 mg/kg at day 5 significantly increased the risk of diarrhea by 1.814 (*P* = 0.002) and 1.622 (*P* = 0.012) folds, respectively, compared to that of the control group. There was no significant risk of diarrhea for other Zn concentrations cross-adjusted with total log_10_CFU. According to treatment types, total log_10_CFU cross-adjusted with ZnA at day 5 significantly increased the risk of diarrhea by 1.448 folds per increments of log_10_CFU units (*P* = 0.006). There was no significant risk of diarrhea in remaining treatment types cross-adjusted with total log_10_CFU. According to Zn concentration, coliform log_10_CFU cross-adjusted with most Zn concentrations at all times significantly increased the risk of diarrhea by 1.628–2.918 folds, except for log_10_CFU cross-adjusted with 500 mg/kg at day 0, and also 500 and 2000 mg/kg at day 20. Based on treatment types, coliform log_10_CFU cross-adjusted with most treatment types at all times significantly increased the risk of diarrhea by 1.804–2.811 folds, except for log_10_CFU cross-adjusted with ZnC at day 0, ZnO and ZnC at day 20.

Overall, the risk of diarrhea due to increase in coliform log_10_CFU was highest in ZnA followed by ZnC, followed by ZnO, and control, except on day 5 where ZnC and ZnO treatment showed similar risks. The risk of diarrhea due to an increase in total log_10_CFU was highest in ZnA although it was only significant on day 5.

### Effect of Zn treatment on the diversity of culturable bacteria

**Fig. 3:**
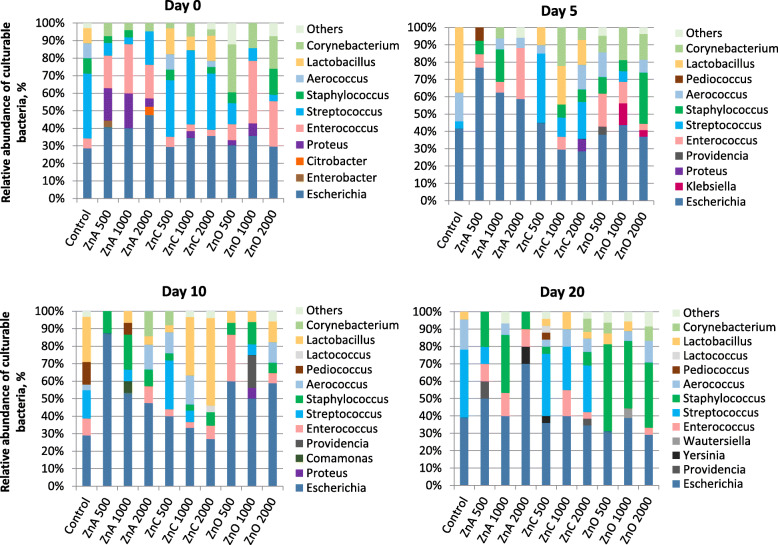
The occurrence of cultivable bacterial genera in piglets feces (identified by MALDI-TOF MS)

The diversity of culturable bacteria was assessed from the same fecal samples taken for evaluation of bacterial concentrations. The results were analyzed as a relative abundance of cultivable bacterial taxa for every piglet group (Fig. 3). *Escherichia* sp. were present in all piglet groups at all time points. Staphylococci were found in piglet feces from all ZnO treatment groups. The ZnA group also showed a higher prevalence of *Streptococcus* sp. and *Lactobacillus* sp. Also, *Enterococcus*, *Aerococcus* and *Corynebacterium* taxa were observed in most of the groups and time points to some extent.

On the day 0, the piglets were weaned and fed with diet containing ZnO and ZnNPs. At that time, the majority of bacteria belonged to *Escherichia* (28.57–47.62%), *Enterococcus* (3.85–35.71%), *Streptococcus* (3.70–42.31%), *Lactobacillus* (0–14.71%) and *Corynebacterium* (0–27.27%) taxa, but *Staphylococcus*, *Aerococcus* and *Proteus* taxa were also present in low abundance. On the 10^th^ day of the experiment *Lactobacillus* sp. (0–50.00%) increased, moreover, the group with the highest concentration of ZnC also showed *Lactococcus* sp. (3.85%). High abundance of *Staphylococcus* sp. (37.50–50.00%) was found in all ZnO concentrations on the 20^th^ day. There were two cases of piglets where *Yersinia enterocolitica* was found (ZnA 2000 mg/kg, and ZnC 500 mg/kg).

### An occurrence of virulence factors in *E. coli*

The 542 of *E. coli* isolates were screened for five virulence genes (*STa*, *STb*, *Stx2*, *F4*, *F18*). These genes have occurred independently or in combinations (Table 4). Only five positive isolates were found at day 0 (two in the control group, two in ZnC 500 mg/kg, one in ZnO 2000 mg/kg). Presence of virulence genes dramatically increased on day 5. All piglets from the control group carried *E. coli* positive for *STb/F4* or *STb/F18*. Moreover, one piglet was positive for *F4* alone. Similar situation was in piglets group fed with ZnA. All piglets from the group ZnA 1000 and 2000 mg/kg diet, and nine from ZnA 500 mg/kg diet were positive for virulence factors. The most common genotype was *STb/F4*. In the case of ZnC 500 mg/kg, there was a high incidence of isolates with virulence genes, mostly *STb/F18*. At a dose of 1000 mg/kg and 2000 mg/kg of ZnC and in the group of ZnO 500 mg/kg, all piglets were negative for all virulence genes screened. In contrast, in groups of ZnO 1000 and 2000 mg/kg, six piglets were positive, primarily for *STb/F4*.

**Table 4:** Virulence genes in *E. coli* isolated from piglet feces

	*STa*	*STb*	*Stx2*	*F4*	*F18*	*STb/F4*	*STb/F18*	*STa/STb*	*Stx2/F18*	*Stx2/STb*	Number of isolates/positive	Positive piglets/piglets with diarrhea
Day 0												
Control	–	1	–	–	–	–	1	–	–	–	15/2	2/0
ZnA 500	–	1/1^a^	–	–	–	–	–	1	–	–	16/2	2/1
ZnA 1000	–	–	–	–	–	–	–	–	–	–	16/0	–
ZnA 2000	–	–	–	–	–	–	–	–	–	–	13/0	–
ZnC 500	–	–	–	–	–	–	–	–	–	–	15/0	–
ZnC 1000	–	–	–	–	–	–	–	–	–	–	12/0	–
ZnC 2000	–	–	–	–	–	–	–	–	–	–	10/0	–
ZnO 500	–	–	–	–	–	–	–	–	–	–	11/0	–
ZnO 1000	–	–	–	–	–	–	–	–	–	–	12/0	–
ZnO 2000	–	–	–	–	–	1	–	–	–	–	16/1	1/0
Day 5												
Control	–	–	–	1	–	5/3 ^a^	5/2 ^a^	–	–	–	12/11	10/5
ZnA 500	–	4	–	1/1 ^a^	–	4/1 ^a^	1	1	–	–	13/11	9/2
ZnA 1000	–	1	–	2/1 ^a^	–	7/4 ^a^	–	1	–	–	12/11	10/5
ZnA 2000	–	3	–	1	–	8/3 ^a^	–	–	–	–	12/12	10/3
ZnC 500	–	–	–	1	–	2	5/2 ^a^	–	–	–	12/8	8/2
ZnC 1000	–	–	–	–	–	–	–	–	–	–	12/0	–
ZnC 2000	–	–	–	–	–	–	–	–	–	–	11/0	–
ZnO 500	–	–	–	–	–	–	–	2	–	–	11/2	2/0
ZnO 1000	–	1	–	–	1	5/3 ^a^	–	–	–	–	10/7	6/3
ZnO 2000	–	–	–	–	–	5/3 ^a^	–	1	–	–	12/6	6/3
Day 10												
Control	–	–	–	–	–	–	–	–	–	–	15/0	–
ZnA 500	–	–	–	–	–	–	7/3 ^a^	–	–	–	8/7	7/3
ZnA 1000	–	–	–	–	–	–	6/2 ^a^	1	–	–	11/7	6/2
ZnA 2000	–	–	–	–	–	4/1 ^a^	3	1	–	–	13/8	6/1
ZnC 500	–	–	–	–	–	–	–	–	–	–	22/0	–
ZnC 1000	–	–	–	–	–	3	–	–	–	–	5/3	3/0
ZnC 2000	–	–	–	–	–	2	–	–	–	–	12/2	2/0
ZnO 500	–	2	–	–	–	–	2	4	–	–	26/8	5/0
ZnO 1000	–	–	–	–	–	–	5	–	–	–	17/5	4/0
ZnO 2000	–	–	–	–	–	1	2	–	–	–	18/3	3/0
Day 20												
Control	–	–	–	–	–	–	–	3	–	–	17/3	2/0
ZnA 500	–	1	–	–	–	–	–	–	–	–	6/1	1/0
ZnA 1000	–	1	–	–	–	–	–	–	–	–	7/1	1/0
ZnA 2000	–	–	–	–	–	–	–	4/2 ^a^	–	–	11/4	2/1
ZnC 500	–	2	–	–	–	–	2	–	–	–	22/4	4/0
ZnC 1000	–	–	–	–	–	2	1	–	–	–	15/3	3/0
ZnC 2000	–	–	–	–	–	2	5/1 ^a^	–	–	–	15/7	6/1
ZnO 500	–	5	2	–	–	–	–	–	2	1	14/10	5/0
ZnO 1000	–	7	–	–	1	–	–	–	–	–	14/8	4/0
ZnO 2000	–	–	–	–	–	–	–	1	–	–	11/1	1/0

^a^ Number of genotypes associated with diarrhea

Compared to day 5, we did not detect any virulence factors in *E. coli* isolates in the control group at day 10 and only two positive piglets were revealed at the day 20. On day 10, there was still relatively high incidence of virulence genes in the group of ZnA (6 or 7 positive piglets in each group) and that decreased on day 20. Interestingly, the dominant genotype was *STb/F18* comparing to that of day 5, where the most isolates were positive for *STb/F4*. Overtime, an occurrence of virulence genes increased in the group of ZnC. In contrast, in ZnA group, the isolates with virulence traits decreased. Piglets in the group of ZnO was mostly positive for *E. coli* with the virulence genes through the entire experiment (Table 4). However, we did not find any correlation between presence of virulence genes and piglet diarrhea.

### Effect of Zn on antioxidant status

**Fig. 4:**
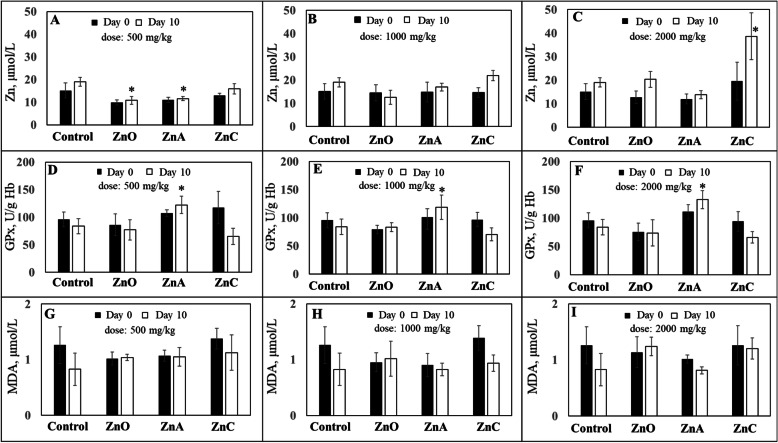
Concentration of zinc (**a**, **b**, **c**); glutathione peroxidase (**d**, **e**, **f**) and malondialdehyde (**g**, **h**, **i**) in blood samples. The applied concentrations of zinc were 500, 1000 and 2000 mg/kg

The significant differences after 10 days of treatment in Zn level and and antioxidant status were observed only in few cases. Compared to the control group, the level of Zn in blood was even lower in treatements ZnO and ZnA at the lowest dose. Significantly different (*P* < 0.05) Zn concentration was detected in piglet blood in the dose of Zn 500 mg/kg diet (Fig. 4a) between groups ZnO (8.3 μmol/L) and ZnA (7.5 μmol/L). At a dose of 1000 mg/kg diet, no significant differences in zinc levels were observed between the treated and control groups (Fig. 4^4^b). In the highest zinc concentration of 2000 mg/kg diet (Fig. 4c), a significant increase of Zn in blood (*P* < 0.05) was observed in the ZnC group (19.6 μmol/L) compared to that of the control group. The changes in antioxidant status (GPx) was found only at ZnA treated group. GPx significantly increased on day 10 (*P* < 0.05) in the ZnA group in diets supplemented with 500, 1000, and 2000 mg Zn/kg diet compared to that of the control group (Fig. 4d-f). The GPx increase in the ZnA group ranged from 35.0 to 48.8 U/g Hb. No significant differences were observed in any other groups (Fig. 4d-f). MDA production was not significantly affected by Zn treatment or concentration in any piglet group (Fig. 4g-i).

**Fig. 5:**
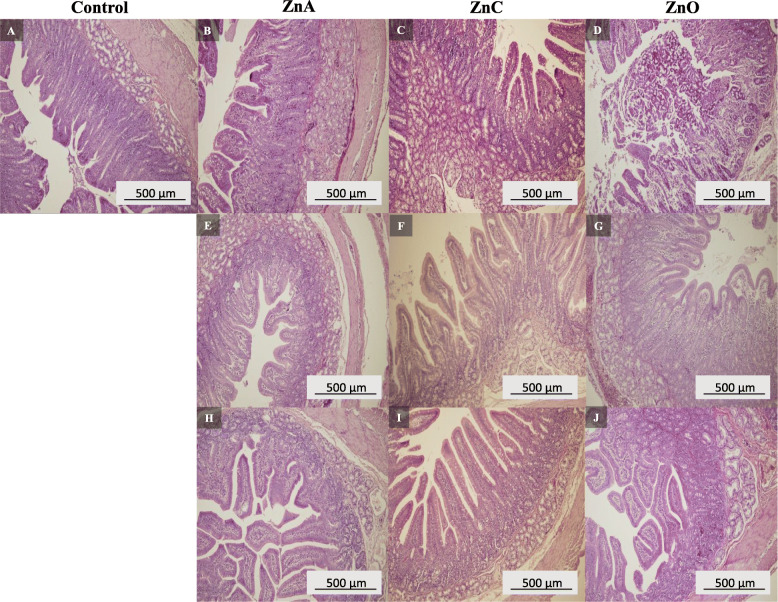
Histological analysis of piglets small intestine. Control (**a**), ZnA 500 mg/kg (**b**), ZnC 500 mg/kg (**c**), ZnO 500 mg/kg (**d**), ZnA 1000 mg/kg (**e**), ZnC 1000 mg/kg (**f**), ZnO 1000 mg/kg (**g**), ZnA 2000 mg/kg (**h**), ZnC 2000 mg/kg (**i**), ZnO 200 mg/kg (**j**)

### Effect of Zn on intestinal and liver morphology

**Fig. 6:**
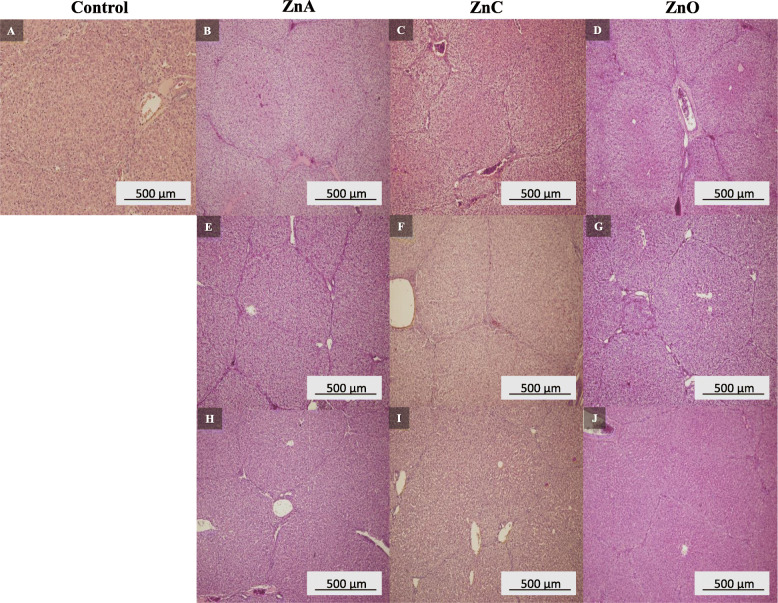
Histological analysis of piglets liver. Control (**a**), ZnA 500 mg/kg (**b**), ZnC 500 mg/kg (**c**), ZnO 500 mg/kg (**d**), ZnA 1000 mg/kg (**e**), ZnC 1000 mg/kg (**f**), ZnO 1000 mg/kg (**g**), ZnA 2000 mg/kg (**h**), ZnC 2000 mg/kg (**i**), ZnO 200 mg/kg (**j**)

Effects of ZnNPs and ZnO treatments on intestinal and liver morphology are shown in Figs. 5 and 6. In the control group, small intestinal inflammations were observed with enlarged spots coinciding with the villi (Fig. 5a). Villlus atrophy occurred only sporadically with a number of Brunner’s glands and malabsorption syndrome was diagnosed. Minimal dystrophic changes were found in liver samples (Fig. 6a). The group ZnA showed chronic enteritis with plaque focal atrophy (Figs. 5b, h) and the malabsorption syndrome. The villi were enlarged and deformed (Fig. 5e). Cuticle and goblet cell showed normal distribution. Liver parenchyma was affected by congestive and full-area dystrophy with portobiliary dilution (Figs. 6b, e, h). The small intestine of the ZnC treated group exhibited inflammatory and atrophic changes, cuticle was in norm (Fig. 5f, i). Mild enteritis and massive incidence of goblet cells have been identified in ZnC treated group at a dose of 500 mg/kg (Fig. 5c). Congestive and dystrophic liver parenchyma (Fig. 6c) as well as dilution of portobilis (Fig. 6f, i) were also noticed. A group treated with ZnO showed changes in the structure manifested by a large number of goblet cells and chronic inflammation without marked alteration of villi (Fig. 5d, g, j). The liver parenchyma in piglets fed with ZnO (Fig. 6d, g) showed chronic inflammation with marked congestion. A full-area glycogen hepatophilia with portobilitary dilatation was observed in the group treated with ZnO at a dosage of 2000 mg/kg (Fig. 6j).

## Discussion

Zinc (in the form of ZnO) is absorbed in the small intestine by simple diffusion and via specific transporters. Zinc enters the small intestinal mucosal cells by binding to histidine, cysteine, and prostaglandins. Inside the intestinal mucosal cell, Zn is bound to the protein metallothionein [24]. Zinc NPs have a higher solubility in the gastrointestinal tract comparing to that of ZnO, and this results in greater antibiotic effect leading to reduction in diarrhea and higher weight gain [12]. The synthesis and characterization of novel hydrogen phosphate-based ZnA and diphosphate-based ZnC NPs were described in our previous work. Moreover, the antibacterial potential and the effect on the microbiota of rats and their overall health was demonstrated [11]. In addition to body weight increase, Zn NPs are able to positively affect the feed conversion ratios [25]. It has been shown that a dose of Zn (as ZnO) of 1200 mg/kg diet is sufficient to improve intestinal integrity and consequently increase the weight gain [26]. In our study, a significant positive effect of ZnA in all concentrations was observed on the weight of weaned piglets. In the dose of 500 mg/kg and 1000 mg/kg of diet, the increment was 3.7 kg, which is comparable with ZnO in the dose of 1000 mg/kg of diet. The highest weight gain, 4.4 kg, was in the group of piglets supplemented with ZnA in the dose of 2000 of mg/kg diet. The use of ZnA in all concetrations approximately doubles weight increments when compared to that of control. This does not fully correlate with the results of a study by Fernandes and colleagues, where the weight of piglets was not affected by Zn dose (from 800 to 2500 mg/kg diet) in the form of ZnO during 21 to 35 days [27]. Wang et al. [13] also demonstrated that ZnO or ZnNPs (size 30 nm) in the dose of 3000 mg/kg diet did not show a significant influence on piglets weight. The body weight gain ranged from 2.54 to 3.09 kg during the experiment [13]. However, our results show a significant increase of weight in piglets fed with ZnO in the dose 1000 mg/kg of diet compared to that of control. In another study, encapsulated zinc was tested at levels of 800 (21 to 35 days of age) and 500 (36 to 49 days of age) mg/kg diet. An average weight loss of 0.37 kg compared to classical ZnO was observed at a dose of 2500 mg Zn/kg diet. A 35.8% increase in diarrhea incidence was observed in the zinc encapsulated group [27]. In our study, we observed the opposite trend, all treatments with ZnNPs led to greater weight gain comparing to that of the control group with the largest increase of weight gain in the ZnA group. It is known that weaning of piglets causes a decrease in the level of zinc and this may exacerbate problems at weaning (diarrhea, weight reduction) [28].

Understanding the composition of the microbial community and its functional capacity during weaning is important for pig production since bacteria play an important role in swine health and growth performance. The total number of culturable bacteria in control, ZnO and ZnNPs groups showed increasing trend of CFUs per gram of feces. Piglets overcome stress caused by dietary changes during the weaning transition through a gut microbiome shift and these results emphasize the importance of the early-life microbiota [29]. Coliform bacteria, milk-fermenting bacteria, and bacteria including *Streptococcus* sp. and *Enterococcus* sp. are found in the gastrointestinal tract after weaning commences [30]. Zinc feed additives therefore seem to have no effect on the presence of the most frequent representatives of coliforms and milk-fermenting bacteria in the gastrointestinal microbiome. There were no obvious differences in the bacterial diversity between piglets on ZnO and ZnNPs, indicating they have similar bactericidal effects. The majority of the bacteria were represented by *Escherichia*, *Enterococcus*, *Streptococcus*, *Lactobacillus* and *Corynebacterium* taxa; however, *Staphylococcus* and *Proteus* sp. were also present. Nevertheless, there were two piglet cases, where *Yersinia enterocolitica* was also found, one of which was presented with bloody diarrhea (day 20, 2000 mg ZnA per kg of feed). In this case, the presence of heat-stable enterotoxin in *E. coli* isolated from the same sample was also confirmed. Another strain of *Y. enterocolitica* was found in the ZnC group (day 20, 500 mg/kg), where the genes for heat-stable enterotoxin and F18 fimbriae in *E. coli* were detected as well. Nevertheless, this piglet did not show any diarrhea symptoms. It is, therefore, evident that the presence of potentially pathogenic bacteria does not necessary lead to diarrhea. However, it is well known that a minimal number of bacterial cells are required for colonization and pathogenicity [31, 32], and our data show quantitative evidence of the role of coliforms in risk of diarrhea (see Additional file Table S2). In most of the studied Zn treatments and concentrations, the increase in coliform CFU has indeed been shown to increase the likelihood of diarrhea. Additionally, it is important to add that diarrhea is also influenced by the overall health status, and it is therefore individual for each animal [33]. For example, Colombo et al. demonstrated that ZnO NPs affect the intestinal barrier. The *in vitro* barrier integrity could be compromised depending on the size and concentration of NPs and the proinflammatory cytokine release was found [34]. Moreover, the oxidative damage of intestinal epithelial cells by ZnO NPs was shown and caused the disarragement of enterocytes’ cytoskeleton and cell junctions’ integrity [35].

Enterotoxigenic *E. coli* (ETEC) are among the most important agents causing post-weaning diarrhea in pigs. ETEC infection is often manifested as watery diarrhea with decrease of weight and eventually death resulting in serious economic losses. Presence of virulence genes in ETEC differs between neonatal and weaned piglets. In post-weaning piglets (PWD), the most common fimbrial antigens are F4 (K88) and F18 [36, 37]. In our study, we detected *E. coli* with these two fimbrial antigens as well. Fimbriae F4 and F18 are responsible for *E. coli* adhesion to the intestinal epithelium and colonization of the small intestine. The most often enterotoxins in PWD are thermostable toxins STa and STb and thermolabile toxin [38, 39]. Our study focused on the detection of STa, STb and on Shiga toxin Stx2 that cause hemorrhagic diarrhea. Our results showed presence of Stx2 only in 4 piglets and none of them was associated with diarrhea. STa and STb were detected mostly in combination with F4, F18 or as STa/STb. It is known that fimbrial genes rarely occur without other virulence traits [40]. In our study, only a few *E. coli* isolates were positive for enterotoxins alone. However, the prevalence of *E. coli* with virulence traits varied over time. The majority of isolates positive for virulence genes was found on the day 5, with the exception of piglets fed ZnC. It is very likely that the piglets at this point were stressed out by weaning as well as environmental and dietary changes which may have led to the increase of ETEC. This was also the time with the highest occurrence of diarrhea correlating with the detection of ETEC. After that, the number of positive isolates decreased in the control and ZnA groups. This indicates that the recovery of normal microbiota in the gut. In piglets in ZnC and ZnO groups, we did not observe any significant changes in the number of ETEC over time. As was mentioned above, presence of ETEC does not necessarily leads to diarrhea. This is in agreement with another study, where ETEC were detected in subclinical infections in pigs [41]. Authors showed that numerous ETEC can persist and circulate in a swine population without clinical manifestation of diarrhea or edema disease. This is important for diagnostics and epidemiology and for understanding the dynamics and ecology of ETEC in pigs.

In the control group, half of the pigs got diarrhea by the day 20 which is high occurrence compared to that of the treated groups. This corresponds with the time the gastrointestinal microbiome was expected to stabilize after weaning. However, samples taken from the control group did not have high prevalence of *E. coli* virulence factors which indicates that diarrhea was caused by other factors.

In previous studies, a higher Zn content in blood serum increased by 6.8 resp. 9.7 mg/mL in weaned piglets fed 450 or 3000 mg Zn/kg diet [12]. In our study, there was an increase in blood Zn concentration at the dose of 2000 mg/kg diet. According to results reported by Pei et al. [12], Zn in ZnNPs was mostly deposited in the pancreas, and the majority of Zn was excreted in the feces. This also in agreement with earlier observations, that showed that linear increase in blood Zn concentration was observed after adding ZnNPs at 15, 30 or 60 mg Zn/kg of diet for 21 days in weaned piglets but other blood parameters were not affected [42]. In another study, the highest increase of Zn concentration in blood plasma and pancreas was observed at a dose of 800 mg ZnNPs/kg of diet [13]. Using ZnNPs at a dose of 1200 mg Zn/kg diet, as in our experiment, a higher final weight was observed when compared to that of the control group. Higher concentrations of zinc in plasma and liver were also observed [43].

As seen from our results, it is necessary to focus not only on the dose of ZnNPs, but also on the modification of nanoparticles. In our study, we used phosphates which may, to some extent, limit the absorption of Zn into the body as also shown in our previous study [11]. At a dose of 2000 mg kg/diet in the diet of rats, the increase in Zn concentration in the blood, liver, and kidney increased compared to that of the control group, and this depended on the type of NPs modification. Other MDA antioxidant parameters, superoxide dismutase (SOD) and oxidized glutathione concentrations were not affected. In another study, the dose of 10 mg ZnNPs/kg of body weight fed to rats for 30 days resulted in higher activity of SOD and GPx in the testicular tissue. In contrast, the MDA level decreased [44]. These results do not entirely correlate with our observation. The antioxidant status of the organism was not significantly affected, except in the ZnA group of piglets where we observed significantly higher GPx activity at the 500, 1000 and 2000 mg/kg diet. ZnNPs were tested *in vitro* using the antioxidant method of 2,2-diphenyl-1-picrylhydrazyl (DPPH), which showed higher efficacy compared to that of the control group and ZnO [45]. Also, Gupta et al. observed a higher antioxidant effect of ZnNPs under *in vitro* conditions using the DPPH assay and Free Radical Scavenging Activities (FRSA) [46]. In contrast to our experiment, these results were obtained under *in vitro* conditions. Also, the form and modifications of ZnNPs were different from those used in our study. In the long-term study (270 days) on mice, the oxidative stress generation by Zn ions and ZnO NPs treatment was not shown. The levels of GSH and MDA in the liver and the lungs did not change compared to that of control. Furthermore, the fecal analysis showed two times higher Zn concentration than that in the control group indicating the main route of Zn excretion [47].

In a previous study, piglets fed by nanozinc (1200 mg/kg diet) showed increased crypt depth, villus length and villus surface area in the ileum and the duodenum compared to that of control (Zn 100 mg/kg diet) [43]. Similar results were obtained in the study of Wang et al. [13], where after supplementation of ZnNPs in the piglet’s diet they observed higher villous height (by 30.9 μm), crypt depth (by 20.2 μm), villous width (by 18.3 μm) and villous surface compared to that of the control group. The addition of 2500 mg/kg diet of ZnO increased the length of the villus height in the jejunum and the ileum by 23 and 22 μm, respectively, compared to that of control receiving 120 mg zinc per kg diet [48]. In our experiment, malabsorption syndrome was diagnosed in the control group and also in the group supplemented by ZnA 2000 mg/kg. This could be due to the combination of the higher zinc dose and the form of ZnNPs based on the hydrogen phosphate precursor. Malabsorption syndrome refers to a number of disorders in which the small intestine cannot absorb enough nutrients and fluids. Nutrients that the small intestine often has trouble absorbing can include macronutrients (proteins, carbohydrates, and fats), micronutrients (vitamins and minerals) or both. Inadequate levels of these nutrients can cause imbalance of antioxidant systems in the body [49]. In the control group of piglets, where the malabsobtion syndrome was found, no zinc blood imbalance was detected. Also, GPx activity and MDA concentrations were not affected in this group. Other studies suggest that the high doses of zinc (2500 mg/kg diet) also affect the enzyme levels in the liver (e.g. arginase1, thiosulfate sulfurtransferase, HSP70), thus supporting the hypothesis of intermediary effects of pharmacological levels of zinc oxide fed to pigs [50]. ZnA and ZnC nanoparticles were tested in a previous *in vivo* experiment and did not show acute toxicity [11].

Due to the forthcoming ban of high pharmacological doses of zinc in the European Union, the efforts are to find alternatives to high-doses of dietary ZnO in pig productions. The dietary supplementation with the low dose (500 mg/kg of diet) of porous Zn and ZnO NPs showed similar or even greater effects on weight gain, intestinal morphology, diarrhea occurrence, and intestine iflamation in piglets compared to that of the high pharmacological dose (3000 mg/kg of diet) of ZnO [51]. Analogously, the dose 600 mg Zn/kg of diet of Zn NPs improved piglets growth performance and reduced diarrhea incidence which is comparable to the high dose of ZnO (2000 mg Zn/kg). The presumed mechanism is intestinal microbiota alteration and inflammation response improvement [52]. However, the fate of the excreted nanoparticles and their effect on the environment remain to be investigated. Clearly, the bioavailability and the toxicity depend on characteristics of different nanoparticles [53–55] and how they interact with the organic matter in the soil [56].

## Conclusion

Two formulations of Zn phosphate-based NPs were tested as alternatives to ZnO, which is traditionaly used as a dietary supplement in livestock productions. The extensive use of high-dose dietary ZnO may result in a long-term adverse effect on the environment, therefore the application of lower-doses ZnNPs with the same effect on swine production could be a potential solution. We demonstrate that dietary supplementations with ZnA NPs significantly increased piglet weight gain even at the lowest concentration (500 mg/kg) without any serious side effects on the piglets. In contrast, the highest ZnO dose (2000 mg/kg) did not have a such effect. Our results indicate that NPs are a promising alternative to high pharmacological doses of conventional ZnO.

## Supplementary information

**Additional file 1: Table S1.** Calculated nutrients; **Table S2.** Factors increasing the risk of diarrhea (cross-adjusted OR by binary logistic regression); **Figure S1.** The temporal comparison of individual groups – ZnA (A, B), ZnC (C, D), ZnO (E, F) and control (G, H) for total counts and coliforms, respectively. Boxes represent Q1 and Q3 quartiles with median bar while T-whiskers represent 95% confidence intervals of 16–30 individuals. * indicates significant differences (*P* < 0.05) between day 0 and 5, 10, 20 day after treatment.

## Data Availability

All data generated or analysed during this study are included in this published article [and its supplementary information files].

## Abbreviations

ANOVA: Analysis of variance
CFU: Colony forming unit
CI: Confidence interval
DPPH: 2,2-diphenyl-1-picrylhydrazyl
ETEC: Enterotoxigenic *E. coli*
FRSA: Free radical scavenging activities
GPx: Glutathione peroxidase
GSH: Glutathione
GSSG: Glutathione disulfide
MALDI-TOF MS: Matrix-assisted laser desorption/ionization time-of-flight mass spectrometry
MDA: Malondialdehyde
NADPH: Nicotinamide adenine dinucleotide phosphate
NPs: Nanoparticles
OR: Odds ratio
PCA: Plate count agar
PWD: Post-weaning piglets
ROS: Reactive oxygen species
SOD: Superoxide dismutase
TBA: Thiobarbituric acid
WG: Weight gain
ZnNPs: Zinc nanoparticles
ZnO: Zinc oxide
