# Supplementary material for: Zinc phosphate-based nanoparticles as alternatives to zinc oxide in diet of weaned piglets
Source: J Anim Sci Biotechnol. 2020 Jun 9;11:59. doi: 10.1186/s40104-020-00458-x (PMC7282173; doi:10.1186/s40104-020-00458-x)

**Additional file**

**Zinc phosphate-based nanoparticles as alternatives to zinc oxide in diet of weaned piglets**

Silvia Kociova^a,b^, Kristyna Dolezelikova^a,b^, Pavel Horky^c^, Sylvie Skalickova^c^, Daria Baholet^c^, Lucie Bozdechova^a,b^, Eva Vaclavkova^d^, Jaroslava Belkova^d^, Pavel Nevrkla^e^ , Jiri Skladanka^c^, Tomas Do^a^, Ondrej Zitka^a,b^, Yazan Haddad^a,b^, Pavel Kopel^a,f^, Ludek Zurek^a,g,h^, Vojtech Adam^a,b^, Kristyna Smerkova^a,b^*^*^*

*^a^Department of Chemistry and Biochemistry, Mendel University in Brno, Zemedelska 1, CZ-613 00 Brno, Czech Republic*

*^b^Central European Institute of Technology, Brno University of Technology, Purkynova 123, CZ-612 00 Brno, Czech Republic*

*^c^Department of Animal Nutrition and Forage Production, Mendel University in Brno, Zemedelska 1, CZ-613 00 Brno, Czech Republic*

*^d^Institute of Animal Science, Pratelstvi 815, CZ-10400 Praha Uhrineves, Czech Republic*

*^e^Department of Animal Breeding, Mendel University in Brno, Zemedelska 1, CZ-613 00 Brno, Czech Republic*

*^f^Department of Inorganic Chemistry, Faculty of Science, Palacky University, 17. listopadu 12, CZ-771 46 Olomouc, Czech Republic*

*^g^Department of Pathology and Parasitology, University of Veterinary and Pharmaceutical Sciences, Brno, Palackeho 1946/1, CZ-612 42 Brno, Czech Republic*

*^h^Central European Institute of Technology, Center for Zoonoses, University of Veterinary and Pharmaceutical Sciences, Brno, Palackeho 1946/1, CZ-612 42 Brno, Czech Republic*

*Corresponding author: Kristyna Smerkova, Department of Chemistry and Biochemistry, Mendel University in Brno, Zemedelska 1, CZ-613 00 Brno, Czech Republic, E-mail: kristyna.smerkova@mendelu.cz; Phone: +420 545 133 293

**Table S1** Calculated nutrients

| **Item** |  | **IU/kg** | **Quantity** |
| --- | --- | --- | --- |
| Dry matter |  | g | 874.1 |
| N- substances |  | g | 179.5 |
| Lysine |  | g | 12.2 |
| Methionine |  | g | 3.9 |
| Sulfur amino acids |  | g | 7.1 |
| Threonine |  | g | 7.9 |
| Tryptophan |  | g | 2.3 |
| Fat |  | g | 34.6 |
| Linoleic acid |  | g | 13.4 |
| Crude fibre  ME - pigs  Ash  Calcium  Phosphorus  Phosphorus available  Natrium  Magnesium  Sulfur  Iron  Manganese  Zinc  Copper  Iodine  Selenium  Vit. A  Vit. D  Tocopherol |  | g  MJ  g  g  g  g  g  g  g  mg  mg  mg  mg  mg  mg  m.j.  m.j.  mg | 39.6  13.5  49.3  7.9  6.5  3.9  2.1  1.4  1.8  297.7  58.4  130.5  127.4  0.6  0.4  10996  1572  135.4 |

^a^ NRC (2014) feed ingredient tables were used for calculation.

**Table S2** Factors increasing the risk of diarrhea (cross-adjusted OR by binary logistic regression).

| **Factor** | **Time** | **Factor** | **Zn Concentration cross-adjusted OR** | | | **Factor** | **Treatment type cross-adjusted OR** | | |
| --- | --- | --- | --- | --- | --- | --- | --- | --- | --- |
|  |  |  | **OR** | **CI (95%)** | ***P*-value** |  | **OR** | **CI (95%)** | ***P*-value** |
| Total CFU | Day 0 | Log_10_CFU × 0 mg | 1 |  |  | Log_10_CFU × None | 1 |  |  |
|  |  | Log_10_CFU × 500 mg | 1.870 | 0.843–4.145 | 0.124 | Log_10_CFU × ZnA | 2.088 | 0.988–4.413 | 0.054 |
|  |  | Log_10_CFU × 1000 mg | 2.211 | 0.997–4.905 | 0.051 | Log_10_CFU × ZnO | 1.817 | 0.857–3.850 | 0.119 |
|  |  | Log_10_CFU × 2000 mg | 2.014 | 0.927–4.375 | 0.077 | Log_10_CFU × ZnC | 1.725 | 0.812–3.664 | 0.156 |
|  | Day 5 | Log_10_CFU × 0 mg | 1 |  |  | Log_10_CFU × None | 1 |  |  |
|  |  | Log_10_CFU × 500 mg | 1.390 | 0.988–1.956 | 0.059 | Log_10_CFU × ZnA | 1.448 | 1.109–1.889 | 0.006* |
|  |  | Log_10_CFU × 1000 mg | 1.814 | 1.240–2.654 | 0.002* | Log_10_CFU × ZnO | 1.200 | 0.902–1.595 | 0.210 |
|  |  | Log_10_CFU × 2000 mg | 1.622 | 1.114–2.361 | 0.012* | Log_10_CFU × ZnC | 1.241 | 0.890–1.731 | 0.203 |
|  | Day 10 | Log_10_CFU × 0 mg | 1 |  |  | Log_10_CFU × None | 1 |  |  |
|  |  | Log_10_CFU × 500 mg | 1.188 | 0.847–1.667 | 0.318 | Log_10_CFU × ZnA | 2.092 | 0.729–5.999 | 0.170 |
|  |  | Log_10_CFU × 1000 mg | 1.367 | 0.979–1.910 | 0.066 | Log_10_CFU × ZnO | 1.778 | 0.623–5.078 | 0.282 |
|  |  | Log_10_CFU × 2000 mg | 1.296 | 0.929–1.808 | 0.126 | Log_10_CFU × ZnC | 1.619 | 0.626–4.188 | 0.320 |
|  | Day 20 | Log_10_CFU × 0 mg | 1 |  |  | Log_10_CFU × None | 1 |  |  |
|  |  | Log_10_CFU × 500 mg | 1.031 | 0.752–1.414 | 0.850 | Log_10_CFU × ZnA | 1.462 | 0.989–2.163 | 0.057 |
|  |  | Log_10_CFU × 1000 mg | 1.273 | 0.953–1.700 | 0.103 | Log_10_CFU × ZnO | 1.199 | 0.819–1.755 | 0.351 |
|  |  | Log_10_CFU × 2000 mg | 1.215 | 0.912–1.620 | 0.184 | Log_10_CFU × ZnC | 1.165 | 0.802–1.692 | 0.424 |
| Coliform CFU | Day 0 | Log_10_CFU × 0 mg | 1 |  |  | Log_10_CFU × None | 1 |  |  |
|  |  | Log_10_CFU × 500 mg | 1.469 | 0.905–2.385 | 0.120 | Log_10_CFU × ZnA | 2.811 | 1.246–6.343 | 0.013* |
|  |  | Log_10_CFU × 1000 mg | 1.770 | 1.101–2.843 | 0.018* | Log_10_CFU × ZnO | 2.119 | 1.050–4.276 | 0.036* |
|  |  | Log_10_CFU × 2000 mg | 1.628 | 1.030–2.573 | 0.037* | Log_10_CFU × ZnC | 2.256 | 0.995–5.117 | 0.052 |
|  | Day 5 | Log_10_CFU × 0 mg | 1 |  |  | Log_10_CFU × None | 1 |  |  |
|  |  | Log_10_CFU × 500 mg | 2.051 | 1.255–3.354 | 0.004* | Log_10_CFU × ZnA | 2.295 | 1.406–3.745 | 0.001* |
|  |  | Log_10_CFU × 1000 mg | 2.918 | 1.692–5.031 | <0.001* | Log_10_CFU × ZnO | 1.885 | 1.183–3.005 | 0.008* |
|  |  | Log_10_CFU × 2000 mg | 2.701 | 1.558–4.682 | <0.001* | Log_10_CFU × ZnC | 1.804 | 1.088–2.990 | 0.022* |
|  | Day 10 | Log_10_CFU × 0 mg | 1 |  |  | Log_10_CFU × None | 1 |  |  |
|  |  | Log_10_CFU × 500 mg | 2.354 | 1.294–4.284 | 0.005* | Log_10_CFU × ZnA | 2.634 | 1.488–4.663 | 0.001* |
|  |  | Log_10_CFU × 1000 mg | 2.702 | 1.542–4.737 | 0.001* | Log_10_CFU × ZnO | 2.225 | 1.259–3.932 | 0.006* |
|  |  | Log_10_CFU × 2000 mg | 2.905 | 1.518–5.557 | 0.001* | Log_10_CFU × ZnC | 2.405 | 1.236–4.678 | 0.010* |
|  | Day 20 | Log_10_CFU × 0 mg | 1 |  |  | Log_10_CFU × None | 1 |  |  |
|  |  | Log_10_CFU × 500 mg | 0.951 | 0.479–1.888 | 0.885 | Log_10_CFU × ZnA | 1.928 | 1.142–3.253 | 0.014* |
|  |  | Log_10_CFU × 1000 mg | 1.732 | 1.012–2.964 | 0.045* | Log_10_CFU × ZnO | 1.289 | 0.757–2.197 | 0.350 |
|  |  | Log_10_CFU × 2000 mg | 1.569 | 0.967–2.548 | 0.068 | Log_10_CFU × ZnC | 1.343 | 0.806–2.238 | 0.257 |

* *P*-value<0.05 is significant.

**Fig. S1** The temporal comparison of individual groups – ZnA (A, B), ZnC (C, D), ZnO (E, F) and control (G, H) for total counts and coliforms, respectively. Boxes represent Q1 and Q3 quartiles with median bar while T-whiskers represent 95% confidence intervals of 16-30 individuals. * indicates significant differences (*P<* 0.05) between day 0 and 5, 10, 20 day after treatment.


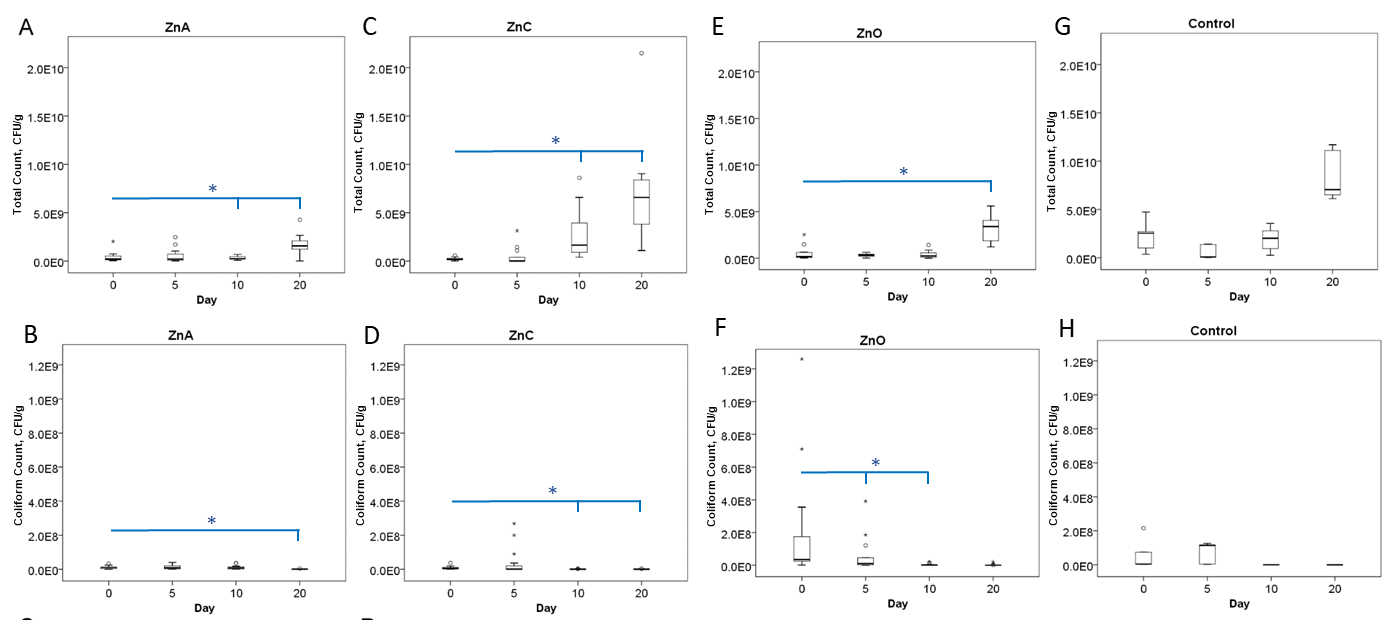

Supplement: Supplementary file 1 — Additional file 1: Table S1. Calculated nutrients; Table S2. Factors increasing the risk of diarrhea (cross-adjusted OR by binary logistic regression); Figure S1. The temporal comparison of individual groups – ZnA (A, B), ZnC (C, D), ZnO (E, F) and control (G, H) for total counts and coliforms, respectively. Boxes represent Q1 and Q3 quartiles with median bar while T-whiskers represent 95% confidence intervals of 16–30 individuals. * indicates significant differences (P < 0.05) between day 0 and 5, 10, 20 day after treatment. [file 40104_2020_458_MOESM1_ESM.docx]
